# The potentiator ivacaftor is essential for pharmacological restoration of F508del-CFTR function and mucociliary clearance in cystic fibrosis

**DOI:** 10.1172/jci.insight.187951

**Published:** 2025-04-22

**Authors:** Anita Balázs, Tihomir Rubil, Christine K. Wong, Jasmin Berger, Marika Drescher, Kathrin Seidel, Mirjam Stahl, Simon Y. Graeber, Marcus A. Mall

**Affiliations:** 1 Department of Pediatric Respiratory Medicine, Immunology and Critical Care Medicine, Charité – Universitätsmedizin Berlin, Berlin, Germany.; 2 German Center for Lung Research (DZL), associated partner site Berlin, Berlin, Germany.; 3 German Center for Child and Adolescent Health (DZKJ), partner site Berlin, Berlin, Germany.; 4 Berlin Institute of Health at Charité – Universitätsmedizin Berlin, Berlin, Germany.

**Keywords:** Cell biology, Pulmonology, Chloride channels, Genetic diseases

## Abstract

Pharmacological rescue of F508del-CFTR by the triple combination CFTR modulator therapy elexacaftor/tezacaftor/ivacaftor (ETI) leads to unprecedented clinical benefits in patients with cystic fibrosis (CF). However, previous studies in CF primary human airway epithelial cultures demonstrated that chronic treatment with the potentiator ivacaftor can render the F508del protein unstable, thus limiting restoration of CFTR chloride channel function. Even so, quantitative studies of this unwanted effect of ivacaftor on F508del channel function with dependency on cell culture conditions remain limited, and the impact of chronic ivacaftor exposure on restoration of mucociliary clearance that is impaired in patients with CF has not been studied. In patient-derived primary nasal epithelial cultures, we found that different culture conditions (UNC-ALI medium vs. PneumaCult medium) have profound effects on ETI-mediated restoration of F508del-CFTR function. Chronic treatment with ivacaftor as part of ETI triple therapy limited the rescue of F508del-CFTR chloride channel function when CF nasal epithelial cultures were grown in UNC-ALI medium but not in PneumaCult medium. In PneumaCult medium, both chronic and acute addition of ivacaftor as part of ETI treatment led to constitutive CFTR-mediated chloride secretion in the absence of exogenous cAMP-dependent stimulation. This constitutive CFTR-mediated chloride secretion was essential to improve viscoelastic properties of the mucus layer and to restore mucociliary transport on CF nasal epithelial cultures. Furthermore, nasal potential difference measurements in patients with CF showed that ETI restored constitutive F508del-CFTR activity in vivo. These results demonstrate that ivacaftor as a component of ETI therapy is essential to restore mucociliary clearance and suggest that this effect is facilitated by its constitutive activation of F508del channels following their folding correction in patients with CF.

## Abstract 2

Ivacaftor is an essential component of triple combination CFTR modulator therapy for pharmacological restoration of F508del-CFTR function and mucociliary clearance in cystic fibrosis airways.

## Graphical abstract

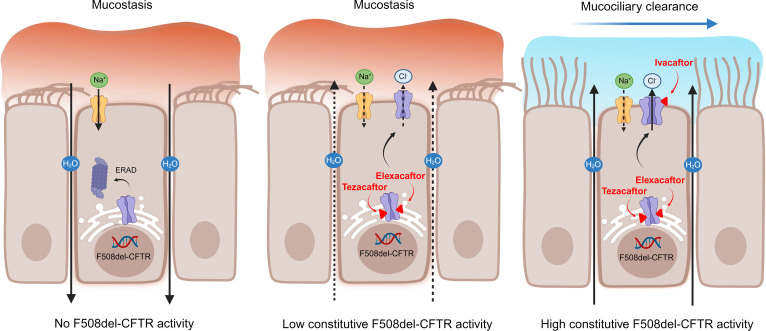

## Introduction

Cystic fibrosis (CF) is a rare hereditary, life-limiting, multiorgan disease caused by mutations in the cystic fibrosis transmembrane conductance regulator (*CFTR*) gene that encodes a cAMP-regulated anion channel. CFTR transports chloride and bicarbonate across the apical membrane of epithelial cells lining the airways and other organ systems of the body, including the gut, pancreas, and reproductive system, where it plays a central role in fluid secretion/hydration and pH regulation of mucosal surfaces and epithelial secretions essential for organ function and homeostasis (1). In the lungs of patients with CF, deficient CFTR-mediated chloride and fluid secretion results in liquid depletion on the airway surface, which causes dehydration/hyperconcentration of the mucus layer representing a key mechanism leading to impaired mucociliary clearance that sets the stage for airway mucus plugging, chronic bacterial infection and inflammation, and progressive lung damage (2–4). Approximately 90% of all patients with CF carry the *F508del* variant on at least 1 allele, making it the most common CF-causing mutation. *F508del* causes multiple molecular defects in the protein, including a severe folding defect leading to mistrafficking and premature intracellular degradation as well as reduced stability and abnormal gating of F508del channels inserted into the cell membrane (5–7). With the emergence of CFTR modulator drugs, it has become possible to address these molecular defects in patients with CF with at least 1 *F508del* allele. The CFTR correctors lumacaftor, tezacaftor, and elexacaftor synergistically increase cellular processing and surface expression, and the potentiator ivacaftor increases the open probability of F508del-CFTR channels expressed at the cell membrane (8–12). Pivotal trials of the currently most effective triple combination CFTR modulator therapy of elexacaftor, tezacaftor, and ivacaftor (ETI) demonstrated unprecedented improvements in clinical outcomes, including pulmonary function and quality of life in CF patients with 1 or 2 *F508del* alleles (13–16). Postapproval observational studies showed that ETI restores CFTR chloride channel function in the airways and intestine to a level of approximately 40%–50% of CFTR function in healthy people (17), improves viscoelastic properties and mucociliary clearance (18, 19), and reduces mucus plugging (20–22) and airway infection and inflammation (18, 23, 24) in adolescent and adult patients with established lung disease.

This successful development of CFTR modulators relied on preclinical testing in patient-derived, highly differentiated airway epithelial cultures that showed a rescue of the F508del processing defect by corrector compounds and potentiation of F508del-CFTR activity upon acute addition of ivacaftor to cultures pretreated with correctors (9, 10). However, patients with CF are continuously treated with fixed dual or triple combinations of CFTR correctors (lumacaftor or tezacaftor or elexacaftor and tezacaftor; ET) and the potentiator ivacaftor (15), and subsequent studies in CF models including primary airway epithelial cultures reported that chronic presence of ivacaftor has adverse effects on the pharmacological correction of F508del-CFTR (25–30). These studies demonstrated that chronic application of ivacaftor in combination with the corrector lumacaftor (25–28), as well as in combination with ET (29, 30), destabilizes F508del-CFTR, thereby limiting its functional rescue. These data suggested that chronic exposure to ivacaftor as part of CFTR modulator combination therapies may interfere with the pharmacological restoration of CFTR function. This raises the possibility that treatment with corrector compounds alone may be more efficacious than a combination therapy including ivacaftor, especially for the corrector combination of tezacaftor with elexacaftor included in elexacaftor/tezacaftor/ivacaftor (ETI), as elexacaftor was reported to have potentiator activity in addition to its synergistic correction of F508del processing (31, 32). However, this possibility has not been tested in clinical trials that included fixed corrector/potentiator combinations only (13–15). Further, quantitative studies of this adverse effect of chronic ivacaftor exposure on rescued F508del channels including a potential influence of cell culture conditions remain limited, and the impact on abnormal viscoelastic properties and mucociliary dysfunction characteristic of CF has not been studied.

The aim of this study was therefore to investigate the acute versus chronic effects of ivacaftor as part of ETI treatment on pharmacological rescue of F508del-CFTR chloride channel function, viscoelastic properties of the mucus layer, and mucociliary transport using different CF cell models and culture conditions. First, we studied the effects of acute versus chronic ivacaftor application on the restoration of CFTR chloride channel maturation and function in an F508del-CFTR–overexpressing cell line by Western blot analysis and electrophysiology. Second, we generated primary nasal epithelial cultures from patients with CF homozygous for the *F508del* mutation, cultured them at air-liquid interface (ALI) in 2 commonly used cell culture media (UNC-ALI medium vs. PneumaCult medium), and studied the impact of acute versus chronic ivacaftor treatment as part of ETI on restoration of F508del-CFTR–mediated chloride secretion by transepithelial bioelectric measurements. Third, we used these highly differentiated CF patient–derived nasal epithelial cultures to investigate the relationship between ivacaftor-dependent effects on F508del-CFTR chloride channel function and the viscosity of the native mucus layer on cultures grown under near-physiological ALI conditions using fluorescent recovery after photobleaching, as well as mucociliary transport (MCT) by tracking the movement of fluorescent beads via time-lapse imaging. Finally, we determined the in vivo relevance of these findings by nasal potential difference measurements in patients with CF before and after initiation of ETI therapy and in healthy individuals.

## Results

### Chronic presence of ivacaftor limits the rescue of F508del-CFTR by ETI in heterologous airway cells.

**Figure 1:**
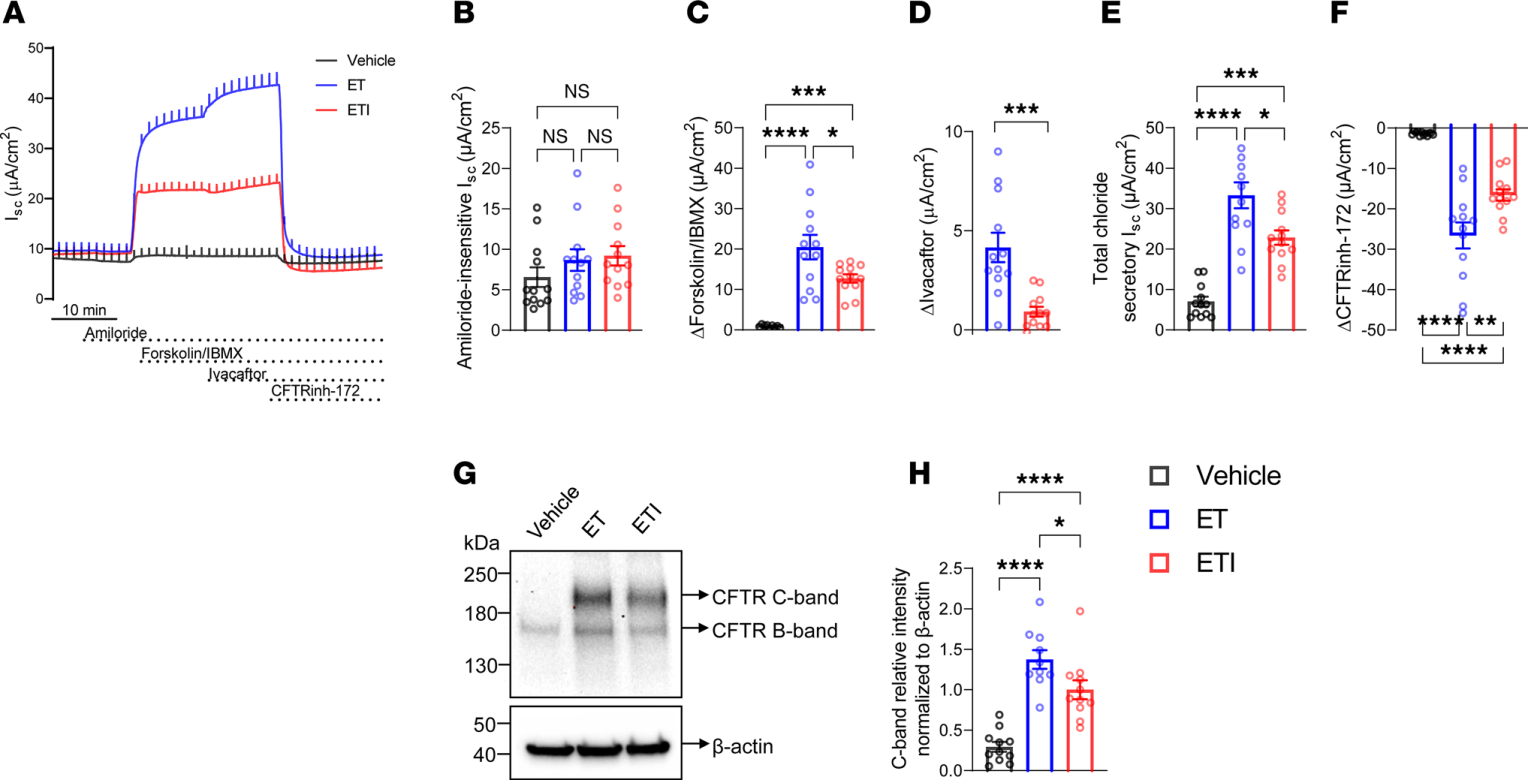
Chronic ivacaftor treatment limits the rescue of F508del-CFTR by ETI in CFBE41o- monolayers. (**A**) Representative original recordings of transepithelial short-circuit current (I_SC_) measurements in CFBE41o- monolayers overexpressing F508del-CFTR pretreated with vehicle alone, elexacaftor and tezacaftor (ET), or elexacaftor/tezacaftor/ivacaftor (ETI) for 24 hours. Ivacaftor was added acutely to ET- or ETI-treated monolayers only. (**B**–**F**) Summary of I_SC_ responses after sequential addition of amiloride, forskolin, ivacaftor, and CFTRinh-172. Total chloride secretory I_SC_ was determined from the sum of amiloride-insensitive I_SC_ and responses to forskolin/3-isobutyl-1-methylxanthin (IBMX) and ivacaftor. *n* = 12 filters per group, and experiments were performed in 5 independent replicates. (**G**) Representative CFTR Western blot with β-actin as loading control. (**H**) Quantification of C-band intensities relative to loading control. *n* = 10–11 samples per group, 3 technical replicates. For statistical testing, 1-way ANOVA with Tukey’s multiple comparison (**B**, **C**, **E**, **F**, **H**) and unpaired *t* test (**D**) were used. *P < 0.05, **P < 0.01, ***P < 0.001, ****P < 0.0001.

To investigate the effects of chronic versus acute ivacaftor exposure on F508del-CFTR correction by ETI treatment, we first tested CF bronchial epithelial cells (CFBE41o-) overexpressing F508del-CFTR, a well-characterized heterologous cell line for CF research (33, 34). Filter-grown CFBE41o- monolayers were pretreated with the CFTR correctors ET alone or in combination with the potentiator ivacaftor (ETI) or vehicle control (DMSO) for 24 hours, and effects on CFTR function were assessed by transepithelial measurements of short-circuit current (I_SC_) in Ussing chambers (Figure 1, A–F). In line with previous reports, inhibition of the epithelial sodium channel (ENaC) with amiloride had negligible effects, indicating its functional absence in this cell line (35). Amiloride-insensitive I_SC_, a parameter that reflects constitutive chloride transport, was comparable between control and both CFTR modulator–treated groups (Figure 1B). Treatment with ET and ETI rescued CFTR function compared with vehicle control, as determined by forskolin/IBMX-stimulated current, total chloride secretion (the sum of amiloride-insensitive current, forskolin/IBMX-stimulated current, and ivacaftor response), as well as CFTRinh-172–inhibited currents. However, chronic presence of ivacaftor markedly decreased CFTR rescue by approximately 40% in ETI- versus ET-treated cultures (Figure 1, C, E, and F). Potentiation of cAMP-stimulated currents by acute addition of ivacaftor was larger in ET- than in ETI-treated cultures (Figure 1D). To study the effects of chronic ivacaftor treatment on F508del-CFTR maturation, we performed immunoblot analysis of whole-cell lysates (Figure 1, G and H). In vehicle-treated cells, F508del-CFTR was detected at around 150 kDa, corresponding to the core glycosylated protein present in the ER (B-band). Treatment with ET and ETI increased the amount of complex glycosylated CFTR detected at around 170 kDa (C-band) compared with vehicle control. However, chronic treatment with ivacaftor decreased the amount of C-band by approximately 30% in ETI- compared with ET-treated cultures.

### Effects of chronic ivacaftor exposure on rescue of F508del-CFTR function by ETI in CF patient–derived nasal epithelia depend on culture conditions.

**Figure 2:**
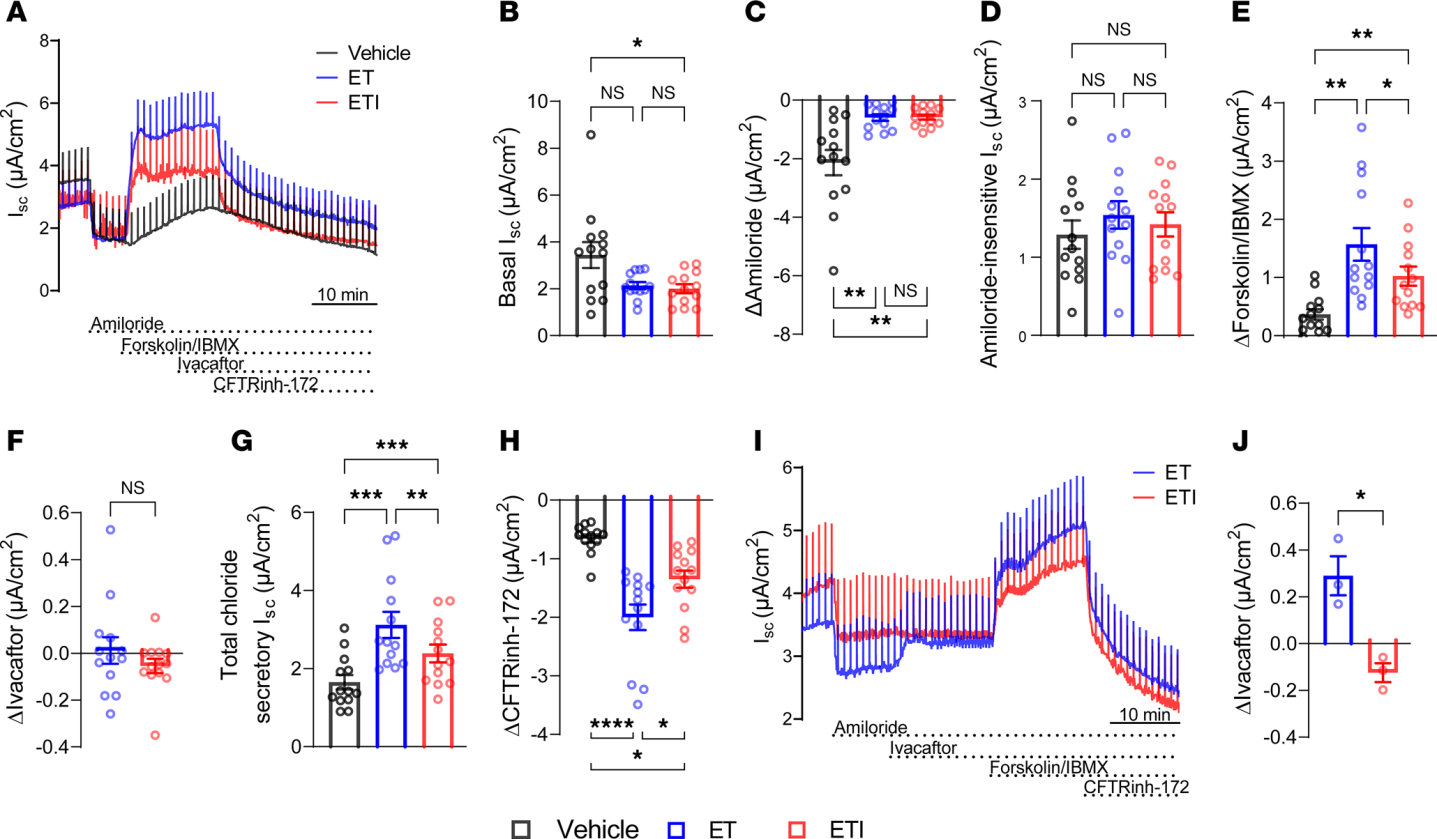
Chronic ivacaftor treatment in combination with elexacaftor and tezacaftor limits the rescue of F508del-CFTR function in CF nasal epithelial cultures grown in UNC-ALI medium. (**A**) Representative original recordings of transepithelial short-circuit current (I_SC_) measurements in primary nasal epithelial cultures derived from CF patients homozygous for *F508del-CFTR*. Cultures were pretreated with vehicle alone, elexacaftor and tezacaftor (ET), or elexacaftor/tezacaftor/ivacaftor (ETI) for 48 hours. Ivacaftor was added acutely to ET- or ETI-treated cultures only. (**B**–**H**) Quantification of I_SC_ responses after addition of amiloride, forskolin/3-isobutyl-1-methylxanthin (IBMX), ivacaftor, and CFTRinh-172. Total chloride secretory I_SC_ was determined from the sum of amiloride-insensitive I_SC_ and responses to forskolin/IBMX and ivacaftor. *n* = 13 donors; data represent mean values of 2 filters per treatment group per patient. (**I**) Representative I_SC_ recordings using a modified Ussing chamber protocol with acute addition of ivacaftor prior to forskolin/IBMX stimulation. (**J**) Quantification of I_SC_ changes after acute administration of ivacaftor in ET- and ETI-treated cultures. *n* = 3 filters per treatment group. For statistical testing, Friedman’s test with Dunn’s multiple comparison (**B** and **H**), 1-way ANOVA with Tukey’s multiple comparison (**C**–**E**, and **G**), Wilcoxon’s test (**F**), and paired *t* test (**J**) were used. *P < 0.05, **P < 0.01, ***P < 0.001, ****P < 0.0001.

To investigate the effects of acute versus chronic ivacaftor treatment in highly differentiated primary airway epithelial cells, we grew nasal epithelial cultures from patients with CF homozygous for *F508del-CFTR* and first studied them under conditions that were previously used in experiments demonstrating that ivacaftor reduces pharmacological rescue in combination with the corrector compound lumacaftor (26). To this end, we expanded nasal epithelial cells by the conditionally reprogrammed cell culture method and differentiated them at ALI using a culture medium developed by Randell et al., termed UNC-ALI (36). Differentiated cultures were treated with ET, ETI, or vehicle alone for 48 hours, and CFTR-mediated chloride transport was assessed in Ussing chambers. Basal I_SC_ was decreased in ETI-treated nasal cultures and showed a trend toward decrease in ET-treated cultures. Treatment with ET and ETI decreased amiloride-sensitive currents compared with vehicle control, indicating a decrease in ENaC-mediated sodium absorption (Figure 2, A–C). Similar to CFBE41o- monolayers, we observed a comparable amiloride-insensitive I_SC_ in all treatment groups (Figure 2D). We detected functional CFTR rescue by both acute and chronic ivacaftor treatment compared with vehicle alone, as determined from forskolin/IBMX-stimulated current, total chloride secretory current, as well as CFTRinh-172–inhibited current (Figure 2, A, E, G, and H). However, in ETI-treated nasal cultures, restoration of CFTR function was lower compared with ET-treated cultures. Acute addition of ivacaftor after cAMP-dependent stimulation by forskolin/IBMX did not further potentiate F508del-CFTR–mediated chloride secretion in ET- or ETI-treated cultures (Figure 2F). In a subset of experiments, we added ivacaftor prior to forskolin/IBMX activation of F508del-CFTR and observed an increase in I_SC_ in ET- but not in ETI-treated cultures (Figure 2, I and J). Taken together, these data show that chronic presence of ivacaftor limits the rescue of F508del-CFTR by ETI in primary nasal epithelial cultures grown in UNC-ALI medium, as previously shown for the dual combination of ivacaftor with lumacaftor (26).

**Figure 3:**
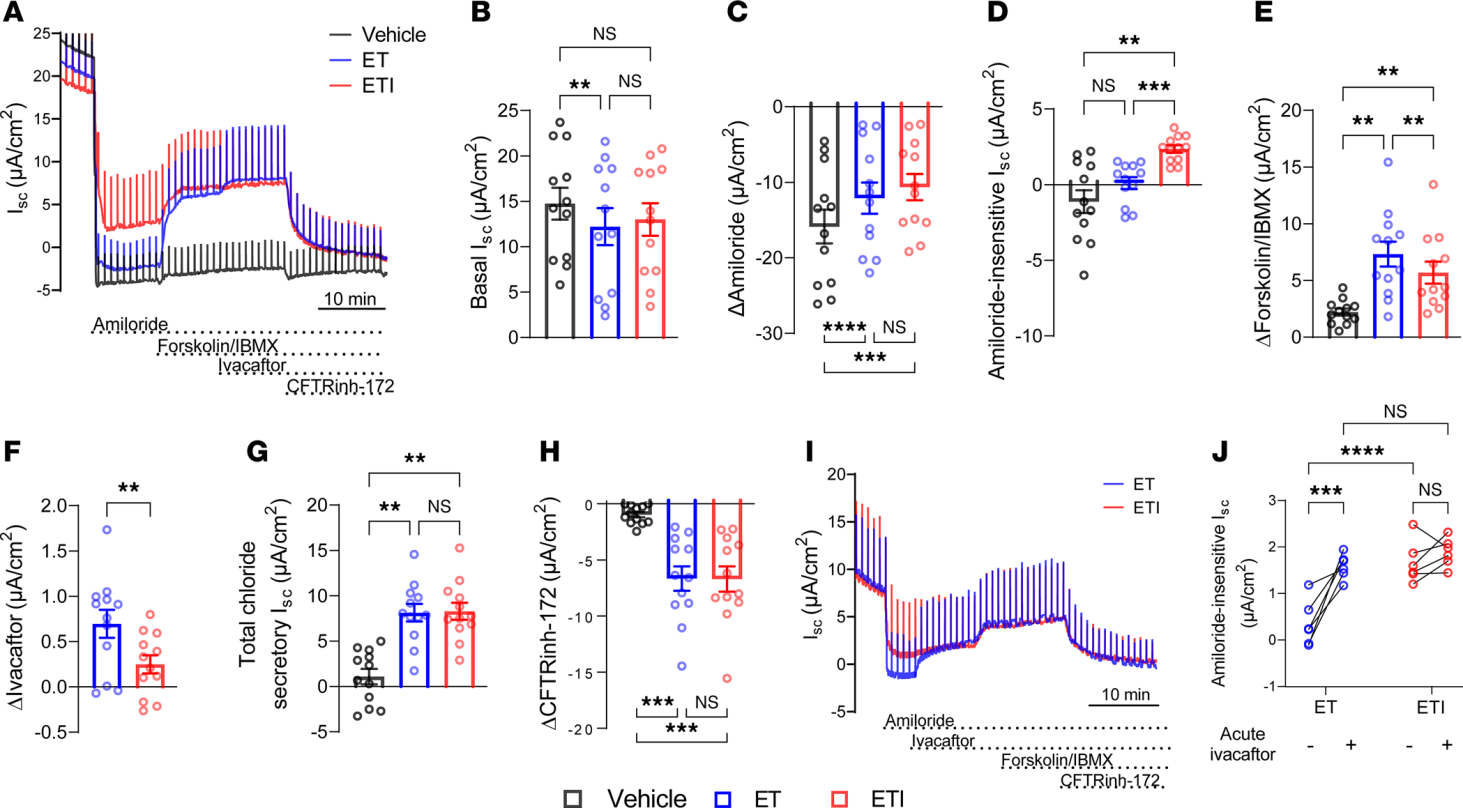
Chronic ivacaftor treatment in combination with elexacaftor and tezacaftor induces constitutive activity of F508del-CFTR in CF nasal epithelial cultures grown in PneumaCult medium. (**A**) Representative original recordings of transepithelial short-circuit current (I_SC_) measurements in primary nasal epithelial cultures derived from CF patients homozygous for *F508del*. Cultures were pretreated with vehicle alone, elexacaftor and tezacaftor (ET), or elexacaftor/tezacaftor/ivacaftor (ETI) for 48 hours. Ivacaftor was added acutely to ET- or ETI-treated cultures only. (**B**–**H**) Quantification of I_SC_ responses after addition of amiloride, forskolin/3-isobutyl-1-methylxanthin (IBMX), ivacaftor, and CFTRinh-172. Total chloride secretory I_SC_ was determined from the sum of amiloride-insensitive I_SC_ and responses to forskolin/IBMX and ivacaftor. *n* = 12 donors; individual data points represent mean values of 2 filters per treatment group per patient. (**I**) Representative I_SC_ recordings using a modified Ussing chamber protocol with acute addition of ivacaftor prior to forskolin/IBMX stimulation. (**J**) Quantification of amiloride-insensitive I_SC_ before and after acute ivacaftor administration in ET- and ETI-treated cultures. *n* = 6 filters per treatment group. For statistical testing 1-way ANOVA with Tukey’s multiple comparison (**B**–**E**, **G**, **H**, and **J**) and paired *t* test (**F**) were used. **P < 0.01, ***P < 0.001, ****P < 0.0001.

Next, we performed similar experiments in nasal epithelial cultures from patients with *F508del* homozygous CF that were differentiated in PneumaCult medium, a proprietary culture medium commonly used to optimize epithelial differentiation, including the formation of cilia by primary airway epithelial cultures (37). Similar to UNC-ALI cultures the basal I_SC_ and the amiloride-sensitive I_SC_ were decreased by both ET and ETI treatment, consistent with decreased activity of ENaC upon CFTR modulator treatment (Figure 3, A–C). In contrast with the results in UNC-ALI cultures, the amiloride-insensitive I_SC_ was significantly higher in ETI-treated compared with ET-treated F508del CF nasal epithelial cells when cultures were grown with PneumaCult medium (Figure 3D). Although both ET and ETI treatment resulted in functional rescue of F508del-CFTR, the cAMP-induced response was smaller in ETI- versus ET-treated cultures, whereas total chloride secretory I_SC_ and CFTRinh-172–inhibited currents were comparable between chronic and acute exposure to ivacaftor (Figure 3, E, G, and H). When viewed in combination, the observed increase in amiloride-insensitive I_SC_, the reduced forskolin/IBMX response, and the comparable magnitude of total chloride secretory I_SC_ and CFTRinh-172–sensitive I_SC_ in ETI-treated compared with ET-treated cultures suggest that chronic exposure to ivacaftor in combination with elexacaftor and tezacaftor induces constitutive activation of F508del-CFTR in CF nasal epithelial cultures grown in PneumaCult medium. This was corroborated in a subset of experiments where CFTRinh-172 was applied directly after amiloride (Supplemental Figure 1; supplemental material available online with this article; https://doi.org/10.1172/jci.insight.187951DS1). Further, comparable total chloride secretory I_SC_ and CFTRinh-172–sensitive I_SC_ after maximal pharmacological stimulation between ET- and ETI-treated cultures indicates that chronic presence of ivacaftor did not limit the functional rescue of F508del-CFTR in CF nasal epithelial cultures grown in PneumaCult medium. Additionally, we found a significantly higher acute ivacaftor response after forskolin/IBMX stimulation in ET- versus ETI-treated cultures (Figure 3F), suggesting that ETI treatment already saturated the cells with ivacaftor, which would be consistent with the high lipophilicity of the drug (28). When F508del CF nasal epithelial cultures were treated acutely with ivacaftor before forskolin/IBMX stimulation, we found that the amiloride-insensitive I_SC_ in ET-treated cultures was increased to the level of ETI-treated cultures (Figure 3, I and J). These results indicate that in the presence of ET, both acute and chronic treatment with ivacaftor induced constitutive activity of F508del-CFTR in CF nasal epithelial cultures grown in PneumaCult medium. To determine whether differences in constitutive F508del-CFTR activity between UNC-ALI– and PneumaCult-grown cultures may be related to differences in intracellular cAMP concentrations, we measured intracellular cAMP in whole-cell lysates, which showed comparable levels between cultures grown in UNC-ALI and PneumaCult media (Supplemental Figure 1).

### Ivacaftor is an essential component of ETI to improve viscoelastic properties of the mucus layer and restore MCT on CF nasal epithelial cultures.

**Figure 4:**
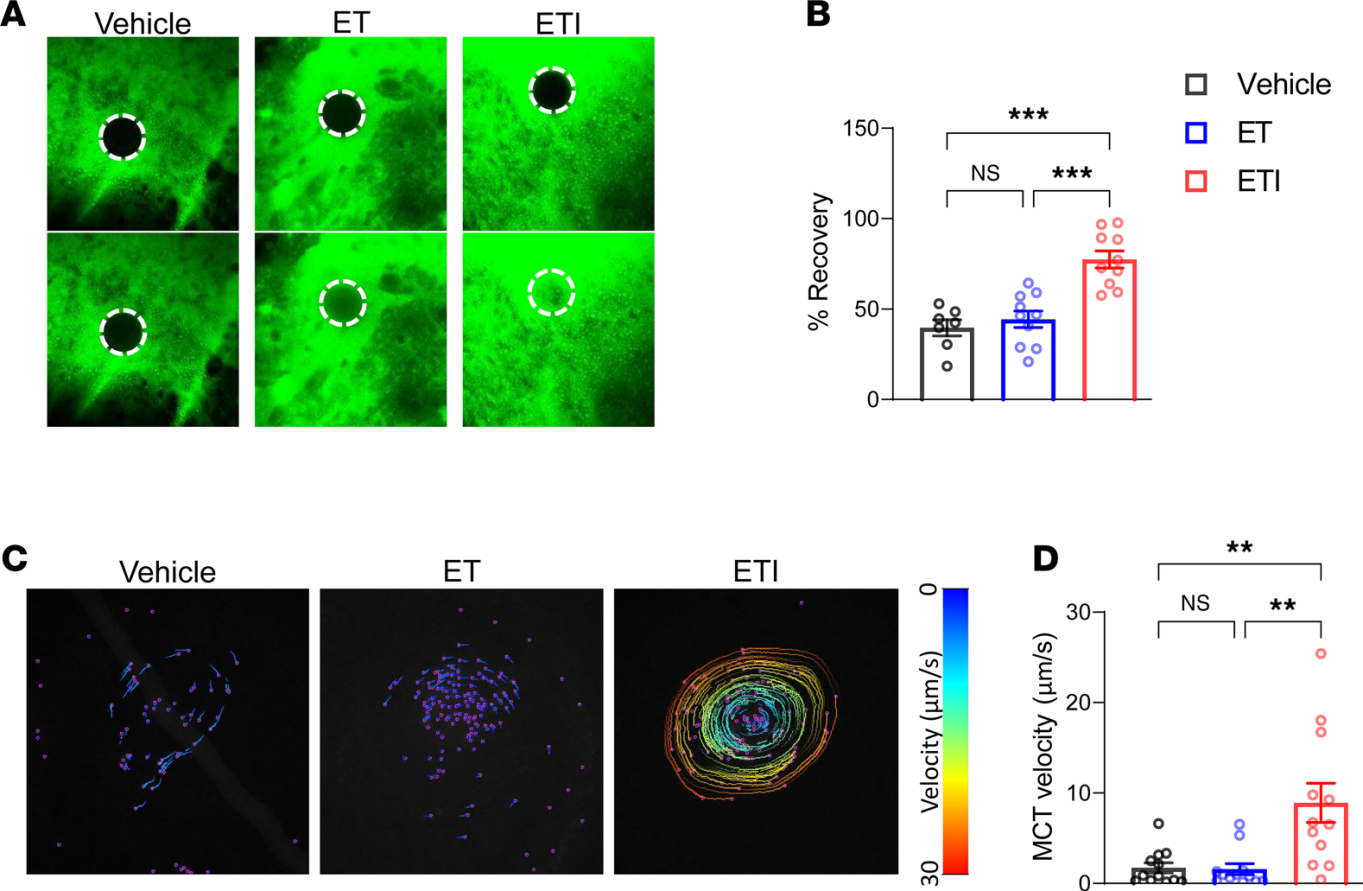
The presence of ivacaftor in combination with elexacaftor and tezacaftor is essential for the improvement of viscoelastic properties of the mucus layer and restoration of mucociliary transport on F508del-expressing CF nasal epithelial cultures. (**A**) Representative images of fluorescent recovery (bottom) after photobleaching (top) of fluorescently labeled mucus on the surface of CF nasal epithelial cultures from *F508del* homozygous CF patients and (**B**) summary of measurements. *n* = 3 donors; 2–3 filters per treatment group per patient. (**C**) Representative bead tracks that visualize mucociliary transport (MCT) velocity determined from transport rates of fluorescent beads added on the surface of nasal epithelial cultures from *F508del* homozygous CF patients and (**D**) summary of measurements. *n* = 3 donors; 4 filters per treatment group per patient. For statistical testing 1-way ANOVA with Tukey’s multiple comparison (**B**) and Kruskal-Wallis test with Dunn’s multiple comparison (**D**) were used. **P < 0.01, ***P < 0.001.

To determine the effect of the ivacaftor-dependent constitutive activation of F508del-CFTR–mediated chloride secretion on the viscoelastic properties of the mucus, we treated CF nasal epithelial cultures grown in PneumaCult medium with ET, ETI, or vehicle control; labeled the mucus layer with fluorescent dye; and measured fluorescent recovery after photobleaching. Treatment with ET alone had no effect on fluorescent recovery of the mucus layer, which reflects diffusion of the fluorescent dye into the bleached area of the mucus gel, whereas fluorescent recovery was significantly increased when CF nasal epithelial cultures were treated with ETI compared with vehicle control (Figure 4, A and B; Supplemental Videos 1–3; and Supplemental Figure 2). Finally, we determined the effect of the presence of ivacaftor on MCT by tracking the movement of fluorescent beads via time-lapse imaging. We found that treatment with ET alone did not facilitate mucus transport, whereas ETI significantly enhanced MCT velocity compared with vehicle control (Figure 4, C and D, and Supplemental Videos 4–6). In a subset of experiments, we added ivacaftor to ET-treated cultures for 24 hours, which increased MCT velocity to similar levels as ETI treatment (Supplemental Figure 3). Collectively, these results indicate that constitutive activation of F508del-CFTR channels by ivacaftor in the presence of the folding correctors ET is essential for improving the viscoelastic properties of the native mucus layer and for restoring MCT on highly differentiated CF primary nasal epithelial cultures.

### ETI therapy increases constitutive CFTR activity in the nasal epithelium of patients with CF and at least 1 F508del-CFTR allele.

**Figure 5:**
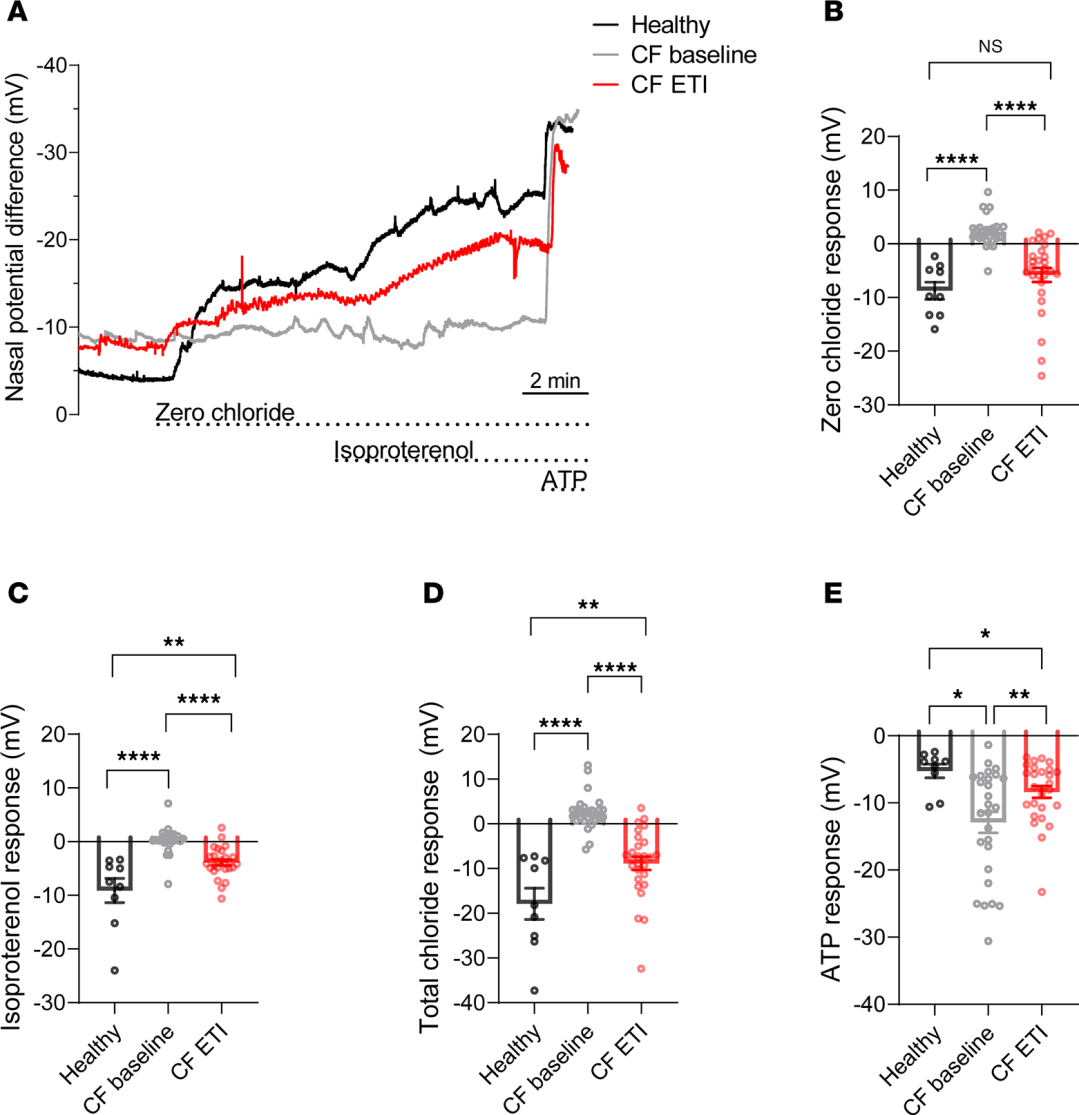
Elexacaftor/tezacaftor/ivacaftor therapy increases constitutive CFTR activity in the nasal epithelium of patients with CF carrying 1 or 2 *F508del-CFTR* alleles. (**A**) Representative nasal potential difference measurement tracings from a healthy individual (black) and a patient with CF at baseline (gray) and after initiation of elexacaftor/tezacaftor/ivacaftor (ETI) therapy (red). (**B**–**E**) Quantification of nasal potential difference responses to zero chloride solution, isoproterenol, total chloride response (determined as the sum of zero chloride and isoproterenol responses), and ATP response in healthy individuals (*n* = 9) and in patients with CF at baseline (*n* = 27) and after initiation of ETI therapy (*n* = 27). Measurements were performed in the presence of amiloride. For statistical testing between CF baseline and CF ETI, paired *t* test or Mann-Whitney test was used as appropriate; between healthy versus CF baseline or CF ETI, unpaired *t* test or Wilcoxon’s test was used as appropriate. *P < 0.05, **P < 0.01, and ****P < 0.0001.

To determine the in vivo relevance of ETI-induced constitutive CFTR activity that we observed in patient-derived airway cultures, we assessed CFTR-mediated chloride secretion in the absence and presence of cAMP-dependent stimulation with isoproterenol as determined by nasal potential difference measurements in healthy individuals and in patients with CF carrying at least 1 *F508del* allele before and after initiation of ETI therapy (17, 38). Before initiation of ETI, as expected from previous studies (38), the change in nasal potential difference observed after superfusion with zero chloride solution in the presence of amiloride reflecting constitutive chloride conductance was significantly reduced in patients with CF compared with healthy individuals (Figure 5, A and B). On ETI therapy, the zero chloride response was significantly increased compared with baseline (Figure 5). Consistent with previous studies (38), responses to subsequent superfusion with isoproterenol to induce cAMP-mediated chloride conductance were also significantly diminished in CF before ETI initiation compared with healthy individuals and were partially restored on ETI therapy (Figure 5, A and C). Similarly, the total chloride response (the sum of zero chloride and isoproterenol responses) was significantly reduced in patients with CF at baseline versus healthy individuals and was partially restored to approximately 50% of the normal values after initiation of ETI therapy (Figure 5, A and D). Finally, stimulation of calcium-activated chloride channels by ATP showed a significantly larger nasal potential difference response in patients at baseline versus healthy people (38), as well as patients at baseline versus on ETI (Figure 5, A and E). Together, these results show that ETI restores both constitutive and cAMP-stimulated F508del-CFTR activity in the nasal epithelium in vivo.

## Discussion

Patient-derived airway epithelial cultures were instrumental in preclinical testing and development of current CFTR modulator therapies targeting the underlying defect in patients with CF (8, 10, 15, 39, 40). However, previous studies in this translational disease model showed that chronic treatment with the potentiator ivacaftor renders the F508del-CFTR protein unstable, limiting the functional restoration of CFTR chloride channels by dual or triple combination CFTR modulator drugs that contain ivacaftor together with the correctors lumacaftor or ET (25, 26, 28–30). Although this effect of chronic ivacaftor exposure may have implications for clinical outcomes in patients with CF, data on a potential role of specific cell culture conditions on the effects of chronic ivacaftor treatment on restoration of F508del-CFTR–mediated chloride secretion are limited, and downstream effects on abnormal viscoelastic properties of airway mucus and impaired mucociliary clearance characteristic of CF have not been studied. This is the first study to our knowledge to demonstrate that the effects of chronic ivacaftor treatment as part of ETI on pharmacological rescue of F508del-CFTR are dependent on the cell context and the growth media. Consistent with previous studies, chronic treatment with ivacaftor limited CFTR-mediated chloride secretion in an F508del-overexpressing cell line (CFBE41o-) and F508del-expressing CF primary nasal epithelial cells cultured with UNC-ALI medium (Figures 1 and 2) (25, 26, 28–30). However, when patient-derived nasal cells were cultured in PneumaCult medium previously shown to support epithelial differentiation, including cilia formation and coordinated cilia-dependent mucus transport (37, 41), we found that the chronic presence of ivacaftor induces constitutive F508del-CFTR activity in the absence of exogenous cAMP-dependent stimulation (Figure 3). This constitutive F508del-CFTR–mediated chloride secretion is essential to improve viscoelastic properties of the mucus layer and to restore MCT on CF airway cultures (Figure 4). These results indicate that ivacaftor is a critical component of ETI that plays a crucial role in improving viscoelastic properties of airway mucus and mucociliary clearance in patients with CF. Moreover, our nasal potential difference measurements in patients with CF support that restoration of constitutive F508del-CFTR function is an important mechanism of ETI-mediated rescue in vivo. These findings have important implications for preclinical testing of CFTR modulators in patient-derived primary airway culture models for CF drug development.

Our data show that the 2 commonly used primary cell culture media (UNC-ALI medium and PneumaCult medium) have substantial and distinct effects on ETI-mediated restoration of F508del-CFTR function in patient-derived CF nasal epithelial cultures. When CF nasal epithelial cultures were grown in UNC-ALI medium, chronic ivacaftor treatment as part of ETI limited the rescue of CFTR function by approximately 30% compared with functional restoration obtained with acute addition of ivacaftor (Figure 2). Similarly, our investigations in the F508del-overexpressing heterologous bronchial epithelial cell line (CFBE41o-) showed that chronic ivacaftor exposure in combination with ET decreased CFTR chloride channel function, paralleled by decreased F508del-CFTR maturation (Figure 1). These observations are in line with previous reports showing that ivacaftor destabilizes F508del-CFTR in the apical membrane, thereby limiting the efficacy of CFTR rescue by corrector compounds lumacaftor or ET (25, 26, 28–30). As expected from the established mode of action, acute ivacaftor administration enhanced F508del-CFTR–mediated chloride secretion both in heterologous cells and in CF primary nasal epithelial cultures, consistent with potentiation of F508del-CFTR channels that are inserted in the plasma membrane following folding correction in the presence of ET. Further, ETI treatment with both acute and chronic administration of ivacaftor led to substantial restoration of CFTR-mediated chloride secretion in different CF cell models used in our study, consistent with the high efficacy of ETI therapy in patients (13–16, 18, 20, 21, 23). In addition, ETI treatment markedly decreased amiloride-sensitive sodium absorption in CF nasal epithelial cultures in both cell culture media, suggesting that pharmacological restoration of CFTR activity may result in ENaC inhibition, as previously demonstrated for coexpression of ENaC with wild-type CFTR but not F508del (42, 43). However, when CF nasal epithelial cells were grown in PneumaCult medium, the magnitude of rescue of CFTR chloride channel function was not affected by the duration of ivacaftor treatment, as indicated by the total chloride secretory current after maximal pharmacological activation as well as inhibition of this current by CFTRinh-172 (Figure 3). Furthermore, both acute and chronic application of ivacaftor induced substantial constitutive CFTR-mediated chloride secretion, prior to cAMP-dependent stimulation (Figure 3 and Supplemental Figure 1). These findings are in contrast with previous studies showing that chronic ivacaftor exposure limits the response to corrector compounds (25, 26, 28, 29) but concordant with other reports of dose-dependent increase in constitutive F508del-CFTR–mediated currents by ivacaftor treatment in combination with ET (29, 44), highlighting the impact of culture conditions on pharmacological rescue of CFTR in CF airway epithelial cultures.

Regarding the constitutive activation of F508del-mediated chloride secretion that we observed with chronic ivacaftor treatment in CF nasal epithelial cultures grown in PneumaCult medium, previous studies on the molecular mechanism of ivacaftor potentiation revealed that it directly binds to F508del-CFTR and induces a phosphorylation-dependent increase in open probability (11, 45–47). In line with this mode of action, increased endogenous cAMP levels were shown to enhance constitutive CFTR activity with chronic presence of ivacaftor in ETI-treated F508del-CFTR–overexpressing CFBE41o- cells, whereas this effect was not observed in ET-treated cells (29). Therefore, we speculate that the constitutive ivacaftor-dependent activation of CFTR function observed in ETI-treated CF nasal epithelial cultures in our study results from its potentiator effects on F508del channels that are inserted into the apical cell membrane and phosphorylated under the control of endogenous cAMP signaling. Further, we speculate this mode of action is similar to the action of ETI in patients, where CFTR phosphorylation also relies on endogenous cAMP signaling, rather than additional cAMP-dependent stimulation that is typically applied in in vitro cell culture studies. This notion is supported by our nasal potential difference measurements demonstrating that ETI therapy improves constitutive and cAMP-induced CFTR activity in the nasal epithelium of CF patients with at least 1 *F508del* allele in vivo (Figure 5). Of note, previous studies reported that the corrector elexacaftor also has potentiator activity (31, 32). However, in our study, treatment of CF cultures with ET alone failed to induce constitutive CFTR-mediated chloride secretion (Figure 3). Moreover, ET alone did not improve mucus viscoelastic properties and failed to restore mucociliary transport on CF cultures (Figure 4). These data suggest that the potentiator potency of elexacaftor is substantially lower than that of ivacaftor in highly differentiated CF nasal cultures.

Impaired mucociliary clearance is a direct consequence of CFTR dysfunction that plays a critical role in the pathogenesis of CF lung disease. Mucociliary dysfunction in CF likely involves multiple mechanisms, including deficient mucus hydration that leads to mucus hyperconcentration because of impaired CFTR-mediated chloride and fluid secretion, as well as abnormal mucin polymer formation in the acidic airway surface pH milieu because of impaired CFTR-mediated bicarbonate secretion (3, 48, 49). Mechanistic studies provided a link between mucus dehydration and mucociliary dysfunction in CF, demonstrating that the increased osmotic pressure of the hyperconcentrated mucus layer leads to compression of the underlying cilia, which impairs efficient cilia-driven transport of mucus (3). To assess the therapeutic relevance of the constitutive ETI-induced and CFTR-mediated chloride secretion observed in CF nasal epithelial cultures grown in PneumaCult medium, we compared mucus viscosity on cultures treated with ET versus ETI alone using fluorescent recovery after photobleaching. We found that chronic presence of ivacaftor was necessary to improve the diffusion of fluorescent particles through the native mucus gel, whereas ET treatment alone did not affect the viscosity of the native mucus layer on CF nasal epithelial cultures compared with vehicle controls. Furthermore, MCT rates on cultures treated with ET alone versus ETI showed that ivacaftor is essential to enhance mucus transport rates, whereas ET-treated cultures failed to transport mucus effectively as observed for vehicle-treated CF cultures (Figure 4). These findings are consistent with recent observational studies that show improved mucus hydration and mucus viscoelastic properties, as well as enhanced mucociliary clearance in patients on ETI therapy (18, 19). Although the relative roles of constitutive versus stimulated CFTR function to coordinate mucociliary clearance have not been fully elucidated in vivo, ex vivo studies in pig bronchioles demonstrate that CFTR conductance is predominantly constitutive (50). Although our transepithelial bioelectric measurements assessed the pharmacological rescue of CFTR function by ETI in the presence of exogenous cAMP agonist, the MCT studies were performed in cultures at ALI under near-physiological conditions in the absence of cAMP stimulation. Under these conditions, CFTR is activated by endogenous cAMP agonists present in the airway surface liquid (e.g., adenosine) (51), which may better reflect in vivo conditions, as supported by our nasal potential difference data in patients treated with ETI (Figure 5). Collectively, our data are consistent with the concept that chronic potentiation by ivacaftor combined with endogenous cAMP phosphorylation of F508del channels that are inserted into the membrane following folding correction by ET produces constitutive F508del-mediated chloride secretion that may play a key role in improving mucus properties and mucociliary clearance in ETI-treated patients with CF.

A limitation of our study is that the mechanisms underlying these culture medium–dependent differences in the responses of CF primary airway cultures to chronic ivacaftor remain unclear, in part because the composition of the PneumaCult medium is proprietary. Both culture media are commonly used in CF research. Although UNC-ALI does not contain any proprietary reagents, PneumaCult has become popular because of its robustness and ease of use. A previous study reported key structural and functional differences of primary airway epithelial cultures comparing these 2 media, including thicker, more ciliated epithelium with PneumaCult, as well as increased expression levels of CFTR and enhanced response to CFTR modulators (37). Further, PneumaCult medium supports the development of coordinated ciliary beating and mucus transport, underscoring its value to recapitulate relevant airway (patho-) physiology (41). This notion is also supported by our results from nasal potential difference measurements showing that by displaying ETI-induced constitutive F508del-CFTR activity, PneumaCult-grown cultures are more representative of the CF airway epithelium in vivo. Although these previously described differences do not explain the unique culture medium–specific differences of ivacaftor effects observed in our study, it is possible that the various mechanisms controlling F508del-CFTR stability and turnover in the apical membrane differ in CF nasal epithelia cultured in PneumaCult versus UNC-ALI medium. For example, differences in endogenous cAMP signaling could represent such a mechanism, which not only regulates the phosphorylation of F508del-CFTR once it has reached the cell surface but also can influence its stability in the apical membrane by the downstream cAMP effector molecule EPAC1 (52–54). It is conceivable that UNC-ALI–grown CF nasal epithelial cultures that do not feature constitutive CFTR-mediated currents lack such positive feedback mechanisms and may therefore not be able to counteract the destabilizing effect of ivacaftor. Of note, we did not find differences in total intracellular cAMP concentrations in nasal epithelial cultures grown in UNC-ALI versus PneumaCult medium (Supplemental Figure 1), and increasing intracellular cAMP levels with submaximal doses of forskolin did not mitigate the detrimental effects of ivacaftor on F508del-CFTR rescue in CFBE41o- cells (Supplemental Figure 4). While these findings argue against a role of global cAMP signaling in the regulation of F508del-CFTR stability, it is possible that local cAMP concentrations in the vicinity of CFTR at the apical membrane may be differentially regulated under different culture conditions (55). Further, we cannot dismiss the possibility that the concentration of free ivacaftor may depend on the composition of cell culture media, which may influence drug exposure of the cultures. These findings also warrant future studies to address the potential limitations of primary cell culture models in their ability to mimic rescue of CFTR function in vivo.

Our data are also relevant for future preclinical testing of novel CFTR modulators and potentially other classes of CFTR-directed therapeutics that are currently being developed to further enhance functional restoration of F508del-CFTR and to restore CFTR function in patients carrying CFTR mutations who are not eligible for ETI (49). For example, our results support that acute addition of ivacaftor to ET-pretreated cultures generates optimized conditions for testing effects on CFTR-mediated chloride secretion in transepithelial bioelectric studies. On the other hand, PneumaCult-grown CF nasal epithelial cultures provide a useful tool to study constitutive activation of F508del-CFTR, as well as its downstream effects on mucociliary dysfunction, which is a key disease mechanism in CF.

In summary, this study shows for the first time to our knowledge that ivacaftor is an essential component of ETI required to induce constitutive activity of F508del-CFTR chloride channels in the absence of exogenous cAMP-dependent stimulation and that this constitutive CFTR-mediated chloride secretion is essential to improve viscoelastic properties of the mucus and restore MCT on nasal epithelial cultures from patients with CF. These findings have important implications for the use of patient-derived airway models for future development of novel CFTR modulators as well as personalized medicine approaches for patients with CF.

## Methods

### Sex as a biological variable.

Participants of this study were of male and female sexes. Sex was not considered as a biological variable, and findings are expected to apply in both sexes.

### Study population.

Studies in primary nasal epithelial cultures included nasal brushings from 14 patients with CF homozygous for the *F508del-CFTR* allele. Nasal epithelial cultures from 13 patients were differentiated in UNC-ALI medium and from 12 patients in PneumaCult medium, and demographics are provided in Table 1. Analyses of nasal potential difference measurements were performed in a subset of healthy volunteers and patients with CF with at least 1 *F508del* allele who were included in a previous multicenter observational study (17). The demographics of participants with nasal potential difference measurements are included in Table 2.

### Heterologous cells.

CF bronchial epithelial cells (CFBE41o-) overexpressing F508del-CFTR (F508del CFBE) were a gift from Eric J. Sorscher (University of Alabama at Birmingham, Birmingham, Alabama, USA) (56). Cells were grown in minimum essential medium supplemented with 10% (v/v) fetal bovine serum, 10 mg/mL glutamine, 100 U/mL penicillin, and 100 μM/mL streptomycin, under puromycin selection at a concentration of 4 μg/mL, which was withdrawn 2–3 passages before endpoint experiments. Cells were trypsinized at 70%–90% confluence, and 500,000 cells were seeded on Snapwell inserts (Corning 3407) and maintained at liquid-liquid interface. The medium was changed every 1–3 days. When transepithelial electrical resistance values reached at least 600 Ω × cm^2^, cells were treated with 0.076% DMSO as vehicle control, 3 μM elexacaftor and 18 μM tezacaftor (ET), or 3 μM elexacaftor, 18 μM tezacaftor, and 1 μM ivacaftor (ETI) for 24 hours (10). All cells were cultured in a humidified incubator at 37°C and in 5% CO_2_. CFTR modulators were obtained from Selleck Chemicals.

### Primary nasal epithelial cultures.

Nasal epithelial cells were obtained noninvasively by nasal brushings and cultured by the conditionally reprogrammed cell culture method as previously described (57–59). After expansion, epithelial cells were seeded at passage 2 or 3 on human placental type IV collagen–coated Snapwell or Transwell (Corning 3407, 3460, or 3470) inserts at a density of 200,000 cells/cm^2^. Nasal epithelial cultures were differentiated at ALI for 4–5 weeks in either UNC-ALI or PneumaCult medium (STEMCELL Technologies). Differentiated nasal epithelial cultures were treated with ET, ETI, or vehicle alone for 48 hours at concentrations described above.

### Ussing chamber measurements.

I_SC_ measurements were performed in EasyMount Ussing chambers (Physiologic Instruments) in voltage clamp configuration as previously described (57, 59). Experiments on CFBE cells were performed in chloride gradient (basolateral 145 mM and apical 5 mM), whereas experiments in primary CF nasal epithelial cultures were performed under symmetric chloride conditions. After 15–20 minutes of equilibration, amiloride (100 μM) was added apically to block ENaC. Next, forskolin (10 μM) and IBMX (100 μM) were applied both apically and basolaterally, followed by addition of ivacaftor (5 μM) (only to ET- or ETI-treated cells) apically, and finally CFTR-inhibitor 172 (20 μM) was added apically to assess CFTR-mediated chloride secretion. In a subset of experiments, ivacaftor was administered before forskolin/IBMX stimulation to study its effect on amiloride-insensitive I_SC_.

### Immunoblot analysis.

Whole-cell lysates of F508del CFBE cells were obtained by scraping the cells from Snapwell filters directly into 80 μL RIPA buffer (Thermo Fisher Scientific) supplemented with 2% sodium dodecyl sulfate and cOmplete protease inhibitor (Merck). After 30 minutes of incubation on ice and occasional vortexing, lysates were cleared by centrifugation at 10,000*g*. For Western blotting, 5 μL of lysates were incubated in Laemmli buffer and 0.05% β-mercaptoethanol at 37°C for 30 minutes and electrophoresed on 4%–20% gradient polyacrylamide gels (Bio-Rad). Proteins were transferred to a PVDF membrane using a Trans-Blot Turbo (Bio-Rad) semidry blotting system according to manufacturer instructions. PVDF membranes were blocked in 5% milk in PBS for 1 hour at room temperature. CFTR was probed with the monoclonal CFTR antibody 596 (provided by John Riordan, University of North Carolina at Chapel Hill, Chapel Hill North Carolina, USA, via the CFTR Antibodies Distribution Program of the Cystic Fibrosis Foundation) at 1:500 dilution overnight at 4°C, followed by an HRP-conjugated goat polyclonal anti-mouse antibody (Dako Denmark, P0047) at 1:5,000 dilution. As loading control, β-actin was probed with monoclonal β-actin antibody (Santa Cruz Biotechnology, sc-477778) at 1:2,000 dilution followed by anti-mouse antibody as described above. Primary and secondary antibodies were incubated in 2.5% milk in PBS with 0.1% Tween 20 at room temperature for 1 hour unless otherwise specified. HRP was detected using the Pierce ECL Western Blotting Substrate (Thermo Fisher Scientific) and imaged with ChemiDoc MP (Bio-Rad) imaging system. Densitometric analysis was performed with FIJI software. To quantify CFTR maturation, the relative amount of CFTR C-band protein was normalized to β-actin loading control, and these levels were used for subsequent calculations (10).

### Intracellular cAMP measurements.

Primary nasal epithelial culture whole-cell lysates were prepared as described above. The protein concentration of the supernatant was measured by a Pierce BCA Protein Assay Kit (Thermo Fisher Scientific), and 200 μg protein was assayed by the cAMP Parameter Assay Kit (R&D Systems, Bio-Techne) as recommended by the suppliers.

### Fluorescent recovery after photobleaching.

To enable efficient in situ photobleaching of fluorescently labeled mucus using an inverted microscope, cells were cultured upside down on the bottom of a filter as previously described (60). Briefly, nasal epithelial cells were inversely seeded on the bottom side of Snapwell inserts overnight at a density of 200,000 cells/cm^2^. After cell adhesion, Snapwells were flipped back to the regular position and cultured at liquid-liquid interface for 2–3 days prior to air lift, when medium was removed from the bottom of the culture dish. Differentiated cultures were treated with ETI, ET, or vehicle as described above. Fluorescent recovery after photobleaching measurements were performed 3 days after treatment. During this time, cultures were not washed to allow mucus accumulation. To label mucus, 2 μL of FITC-conjugated 2 MDa dextran (2 μg/mL, Thermo Fisher Scientific) was added to the apical side. As cilia-driven mucus transport may influence the diffusion of microparticles and thereby interfere with fluorescent recovery after photobleaching measurements, we applied cinnamaldehyde (1:1,000) to the basolateral medium, 30 minutes prior to imaging, to inhibit coordinated ciliary movement and MCT as previously described (61). Confocal imaging was performed using a Leica Stellaris 8 confocal laser scanning microscope with fluorescent recovery after photobleaching modality. Time-lapse videos were recorded for 5 frames (1.29 s/frame) before and 120 frames after 30 seconds of photobleaching of a preselected circular region of interest (ROI). Three ROIs per filter were evaluated. Fluorescent recovery after photobleaching images were processed and analyzed using FIJI with Stowers plugin (62). Fluorescence intensity values were normalized to baseline, and endpoint intensities were determined as percentage recovery.

### MCT velocity measurements.

Highly differentiated nasal epithelial cultures grown at ALI were washed with PBS for 15 minutes twice to remove mucus. Fluorescent carboxylate-modified polystyrene beads (FluoSpheres, Thermo Fisher Scientific) conjugated to Nile red dye with 2 μm diameter were diluted in PBS (~660 beads/μL) and added to the cultures apically at a density of 1,500 beads/cm^2^. Nasal epithelial cultures were then treated with vehicle, ET, or ETI as described above, and MCT was investigated 3 days after treatment or later, if indicated otherwise. During this time, cultures were not washed to allow mucus accumulation. Wide-field, fluorescent, time-lapse imaging was performed using a Stellaris 8 confocal laser scanning microscope equipped with Hamamatsu Orca Flash 4.0 V3 scientific CMOS camera. Images were acquired with a 2.5× air objective with a Texas red filter every second for 1 minute. Image analysis was performed with the FIJI Trackmate plug-in (63).

### Nasal potential difference measurements.

Nasal potential difference measurements were performed in a previous prospective multicenter observational study (17) as previously described (64). Analyses of nasal potential difference measurements, including the response to superfusion with zero chloride and isoproterenol to determine the constitutive and cAMP-induced chloride conductance, respectively, were performed in a subset of 9 healthy volunteers and 27 patients with CF with 1 or 2 *F508del* alleles at baseline and 8–16 weeks after initiation of ETI therapy.

### Statistics.

Data were analyzed with Prism 9.5.1 for Windows (GraphPad Software). Anderson-Darling test was used to assess normality of data distribution. Two-group comparisons were performed with 2-tailed paired *t* test, Wilcoxon’s test, or Mann-Whitney test as appropriate, and multiple-group comparisons were performed using 1-way ANOVA followed by Tukey’s multiple-comparison test or Kruskal-Wallis test or using Friedman’s test with Dunn’s multiple-comparison test as appropriate and indicated in each figure legend. *P* < 0.05 was accepted to indicate statistical significance. All data are presented as mean ± SEM.

### Study approval.

The study was approved by the ethics committee of the Charité – Universitätsmedizin Berlin (EA2/015/18 and EA2/220/18). Written informed consent was obtained from all patients.

### Data availability.

All data are available in the main text or the supplemental materials, including the Supporting Data Values file.

## Author contributions

AB, TR, and MAM conceived the study. AB, TR, CKW, JB, KS, MD, SYG, and MAM developed methodology. AB, TR, CKW, JB, KS, MD, MS, SYG, and MAM investigated. AB, TR, CKW, JB, and MAM visualized data. MAM acquired funding and supervised the study. AB, TR, and MAM wrote the original draft. CKW, JB, KS, MD, MS, and SYG reviewed and edited the draft. AB and TR are co–first authors, with AB listed first, as she initiated the project.

## Supplementary Material

Supplemental data

Unedited blot and gel images

Supplemental video 1

Supplemental video 2

Supplemental video 3

Supplemental video 4

Supplemental video 5

Supplemental video 6

Supporting data values

## Figures and Tables

**Table 1:**
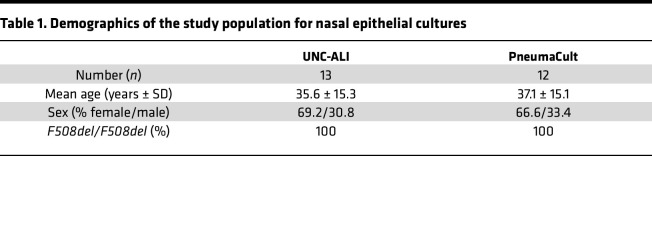
Demographics of the study population for nasal epithelial cultures

**Table 2:**
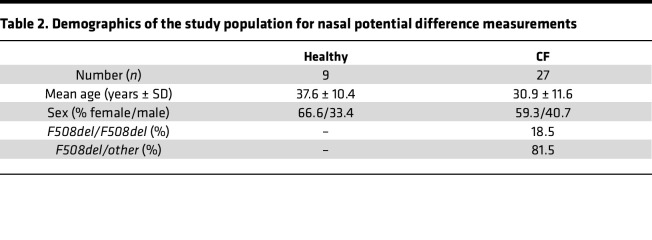
Demographics of the study population for nasal potential difference measurements

## Version 1 04/22/2025

In-Press Preview

## Version 2 05/22/2025

Electronic publication
