# Supplementary material for: The potentiator ivacaftor is essential for pharmacological restoration of F508del-CFTR function and mucociliary clearance in cystic fibrosis
Source: JCI Insight. 2025 Apr 22;10(10):e187951. doi: 10.1172/jci.insight.187951 (PMC12128954; doi:10.1172/jci.insight.187951)
Supplement: Supplemental data [file jciinsight-10-187951-s051.pdf]

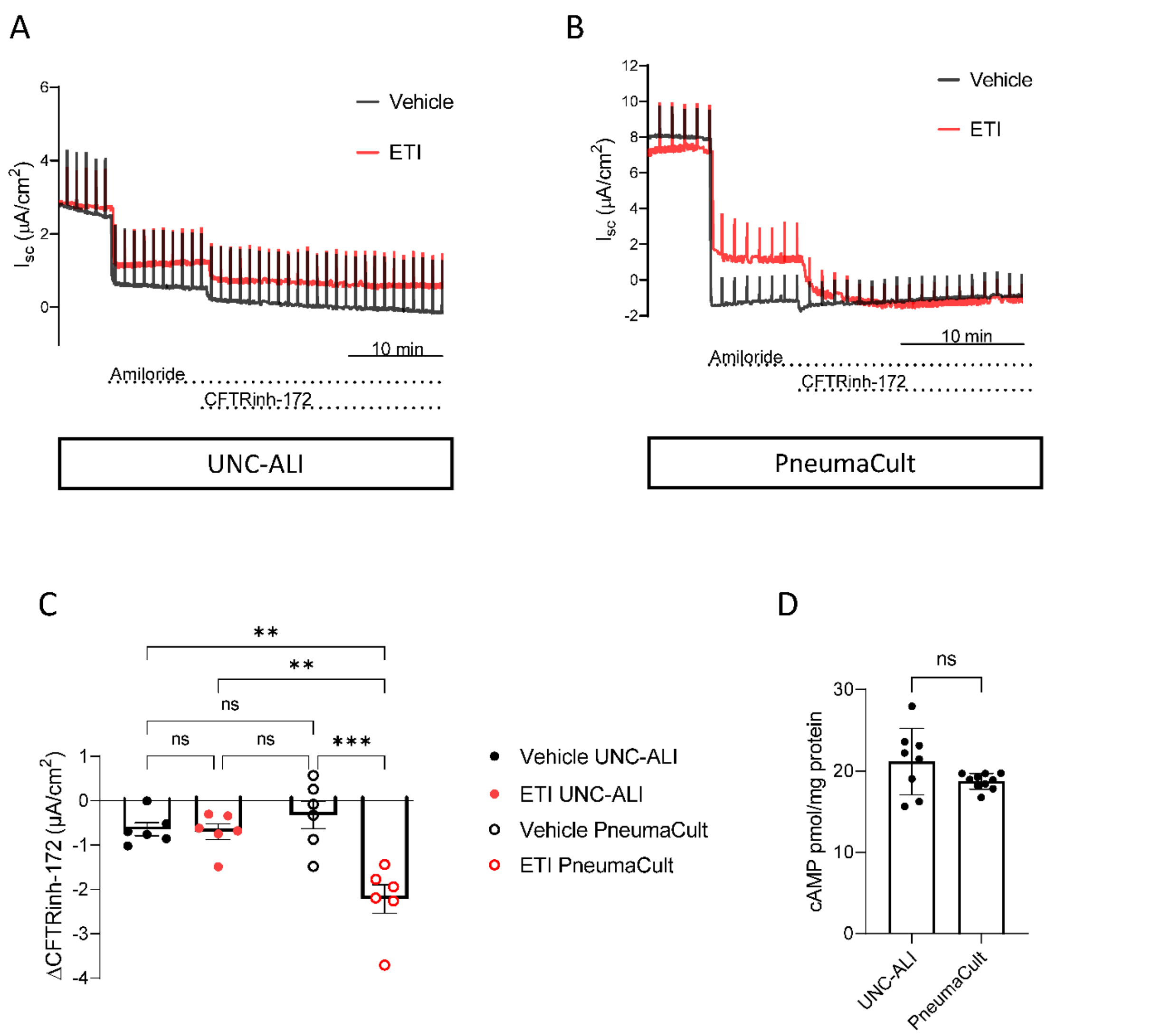

Figure S1. Chronic ivacaftor treatment in combination with elexacaftor and tezacaftor induces constitutive activity of F508del-CFTR in CF nasal epithelial cultures grown in PneumaCult but not in UNC-ALI medium. (**A, B**) Representative original recordings of transepithelial short-circuit current (I_{sc}) measurements in primary nasal epithelial cultures derived from CF patients homozygous for F508del grown in (A) UNC-ALI medium or (B) PneumaCult medium. Cultures were pre-treated with vehicle alone or elexacaftor/tezacaftor/ivacaftor (ETI) for 48 h. (**C**) Quantification of I_{sc} responses after addition of CFTRinh-172. n = 6 donors. (**D**) Intracellular cAMP measurement in whole cell lysates of primary nasal epithelial cultures derived from CF patients homozygous for *F508del* grown in UNC-ALI medium or PneumaCult medium. n = 8-10 donors. For statistical testing one-way ANOVA with Tukey´s multiple comparison (C) or unpaired t test (D) were used. **p<0.01, ***p<0.001.

A

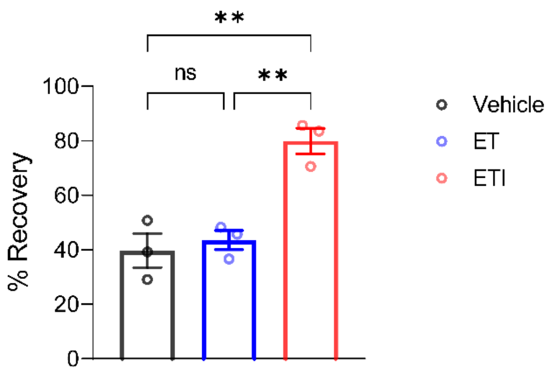

B

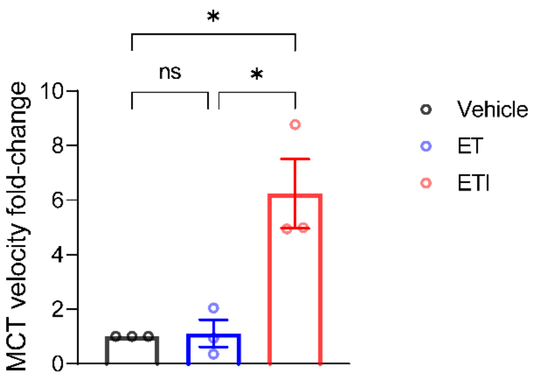

Figure S2. Effect of ivacaftor in combination with elexacaftor and tezacaftor on the viscoelastic properties of the mucus layer and mucociliary transport on F508del-CFTR expressing CF nasal epithelial cultures from individual donors. (**A**) Fluorescent recovery after photobleaching (FRAP) of fluorescently labeled mucus on the surface of CF nasal epithelial cultures from F508del homozygous CF patients. n = 3 donors, data points represent mean values of 2-3 filters per treatment group per patient. For statistical testing one-way ANOVA with Tukey´s multiple comparison was used. $p < 0.01$. (**B**) Mucociliary transport (MCT) velocity fold-changes determined from transport rates of fluorescent beads added on the surface of nasal epithelial cultures from F508del homozygous CF patients. Data were normalized to vehicle control. n = 3 donors, data points represent mean values of 4 filters per treatment group. For statistical testing one-way ANOVA with Tukey´s multiple comparison was used. $p < 0.05$.

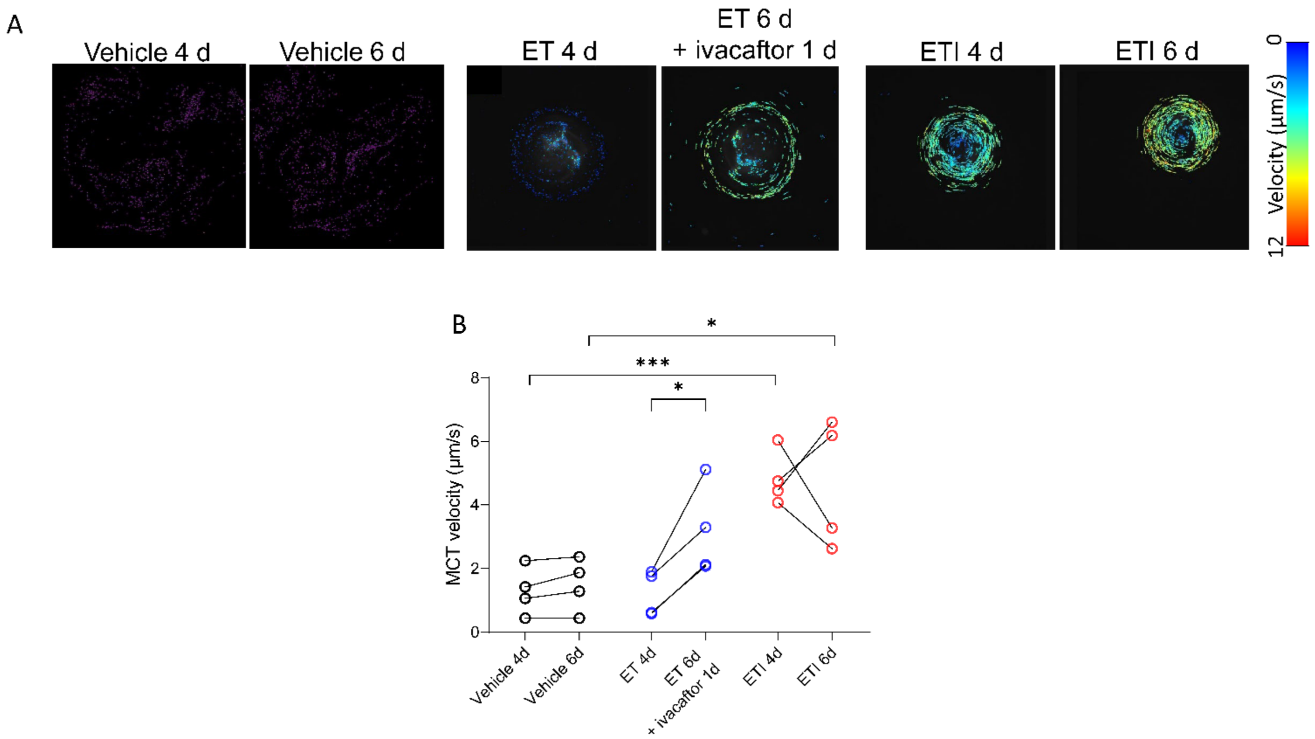

Figure S3. Addition of ivacaftor restores mucociliary transport on F508del-expressing CF nasal epithelial cultures pre-treated with ET. (**A-B**) Longitudinal measurements of mucociliary transport (MCT) velocity determined from transport rates of fluorescent beads added on the surface of nasal epithelial cultures from a *F508del* homozygous CF patient. Cultures were treated with vehicle alone, elexacaftor/tezacaftor (ET) or elexacaftor/tezacaftor/ivacaftor (ETI). Representative bead tracks that visualize MCT velocity at day 4 and day 6 after treatment initiation (A), and summary of measurements (B). n = 4 filters per treatment group. For statistical testing paired t test (B) and one-way ANOVA with Tukey´s multiple comparison were used (B). *$p<0.05$, ***$p<0.001$.

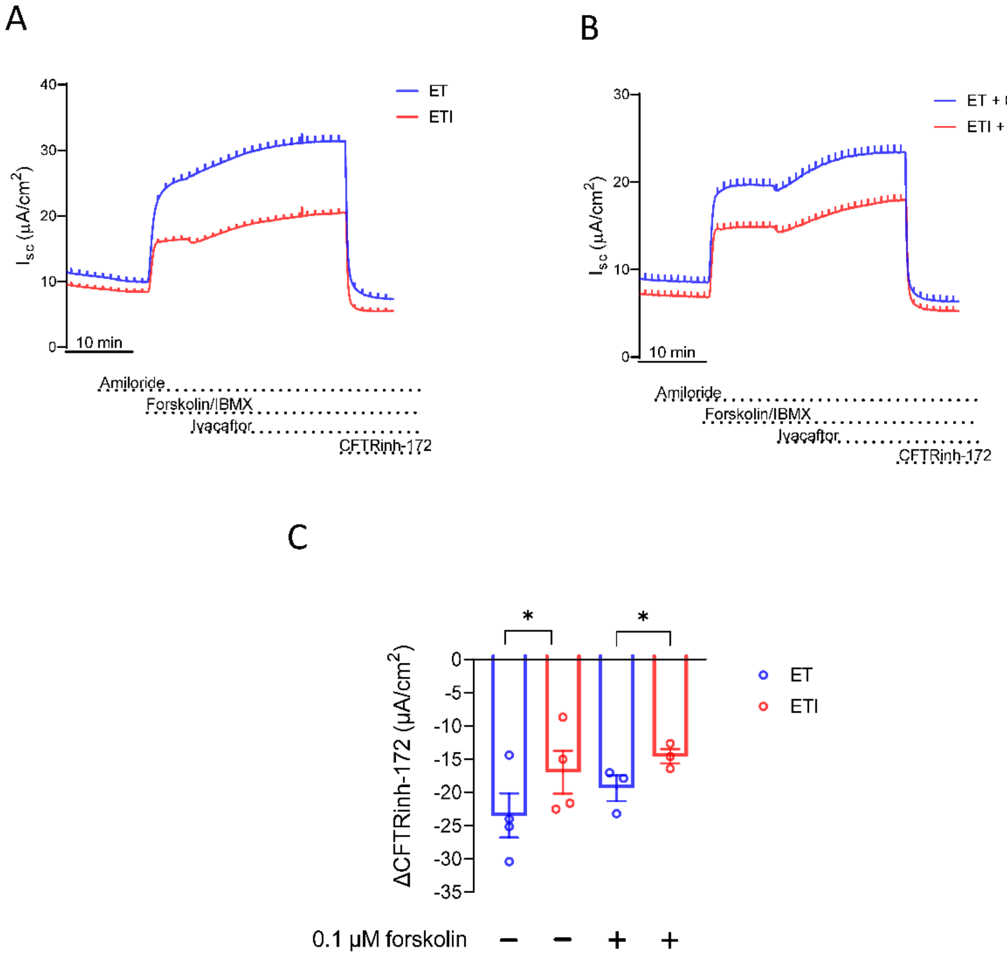

Figure S4. Chronic treatment with forskolin does not affect the limiting effects of ivacaftor on the rescue of F508del-CFTR by ETI in CFBE41o- monolayers. (**A, B**) Representative original recordings of transepithelial short-circuit current (I_{sc}) measurements in CFBE41o- monolayers overexpressing F508del-CFTR pre-treated with vehicle alone, elexacaftor/tezacaftor (ET) or elexacaftor/tezacaftor/ivacaftor (ETI) for 24 h in the (A) absence or (B) presence of 0.1 µM forskolin. (**C**) Summary of I_{sc} responses to CFTRinh-172. n = 3-4 filters per group, experiments were performed in independent replicates. For statistical testing paired t test was used. *$p<0.05$.

Supplemental Videos

1: Time-lapse imaging of fluorescent recovery after photobleaching (FRAP) of fluorescently labeled mucus on the surface of CF nasal epithelial cultures from F508del homozygous CF patients treated with vehicle.

2: Time-lapse imaging of fluorescent recovery after photobleaching (FRAP) of fluorescently labeled mucus on the surface of CF nasal epithelial cultures from F508del homozygous CF patients treated with ET.

3: Time-lapse imaging of fluorescent recovery after photobleaching (FRAP) of fluorescently labeled mucus on the surface of CF nasal epithelial cultures from F508del homozygous CF patients treated with ETI.

4: Time-lapse imaging of mucociliary transport (MCT) velocity determined from transport rates of fluorescent beads added on the surface of nasal epithelial cultures from F508del homozygous CF patients treated with vehicle.

5: Time-lapse imaging of mucociliary transport (MCT) velocity determined from transport rates of fluorescent beads added on the surface of nasal epithelial cultures from F508del homozygous CF patients treated with ET.

6: Time-lapse imaging of mucociliary transport (MCT) velocity determined from transport rates of fluorescent beads added on the surface of nasal epithelial cultures from F508del homozygous CF patients treated with ETI.
