# Supplementary material for: The potentiator ivacaftor is essential for pharmacological restoration of F508del-CFTR function and mucociliary clearance in cystic fibrosis
Source: JCI Insight. 2025 Apr 22;10(10):e187951. doi: 10.1172/jci.insight.187951 (PMC12128954; doi:10.1172/jci.insight.187951)

Blot I. (representative)

CFTR antibody 596

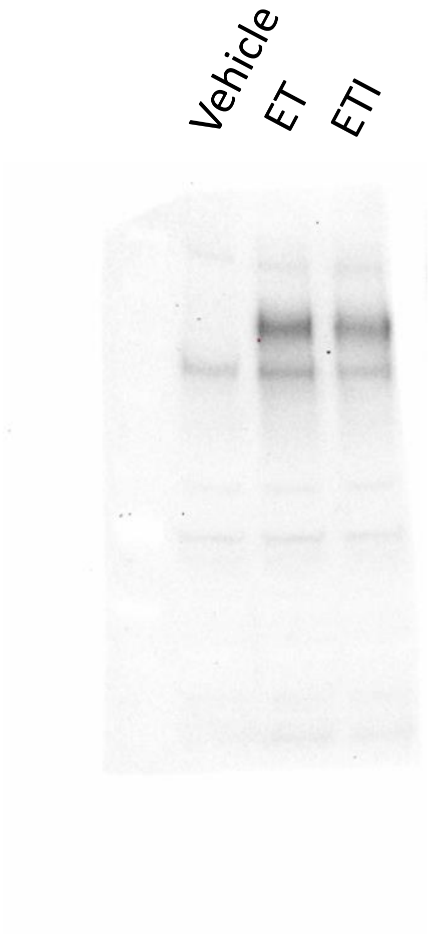

β-actin antibody

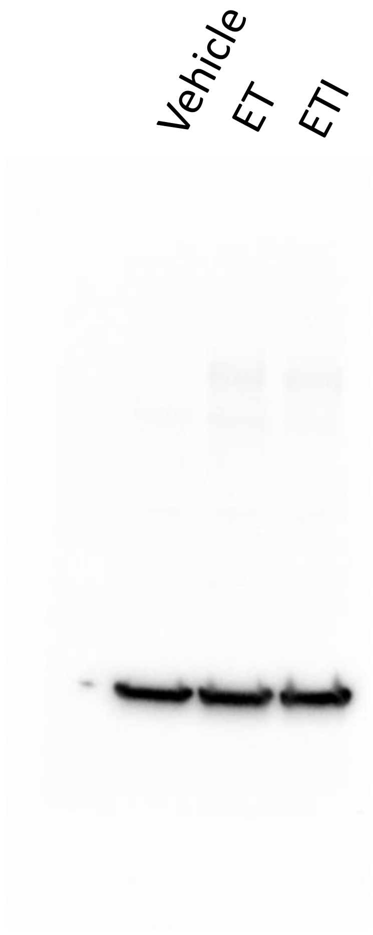

Blot II.

CFTR antibody 596

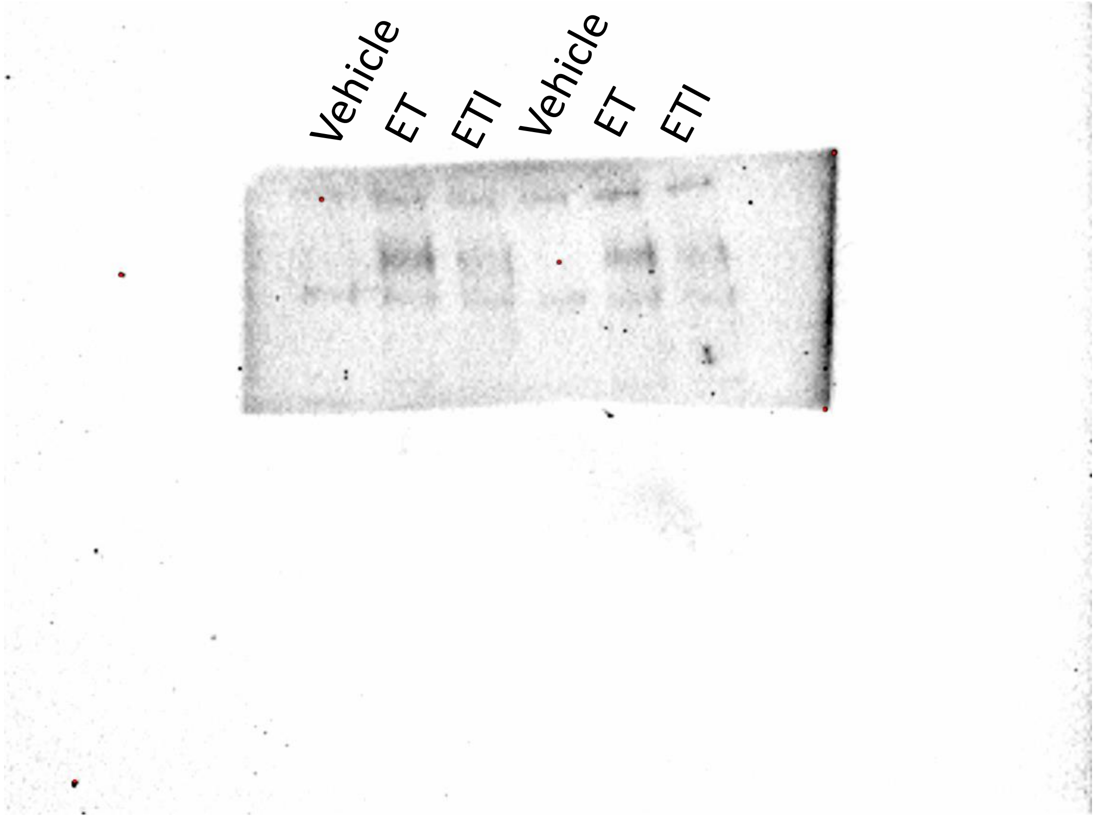

β-actin antibody

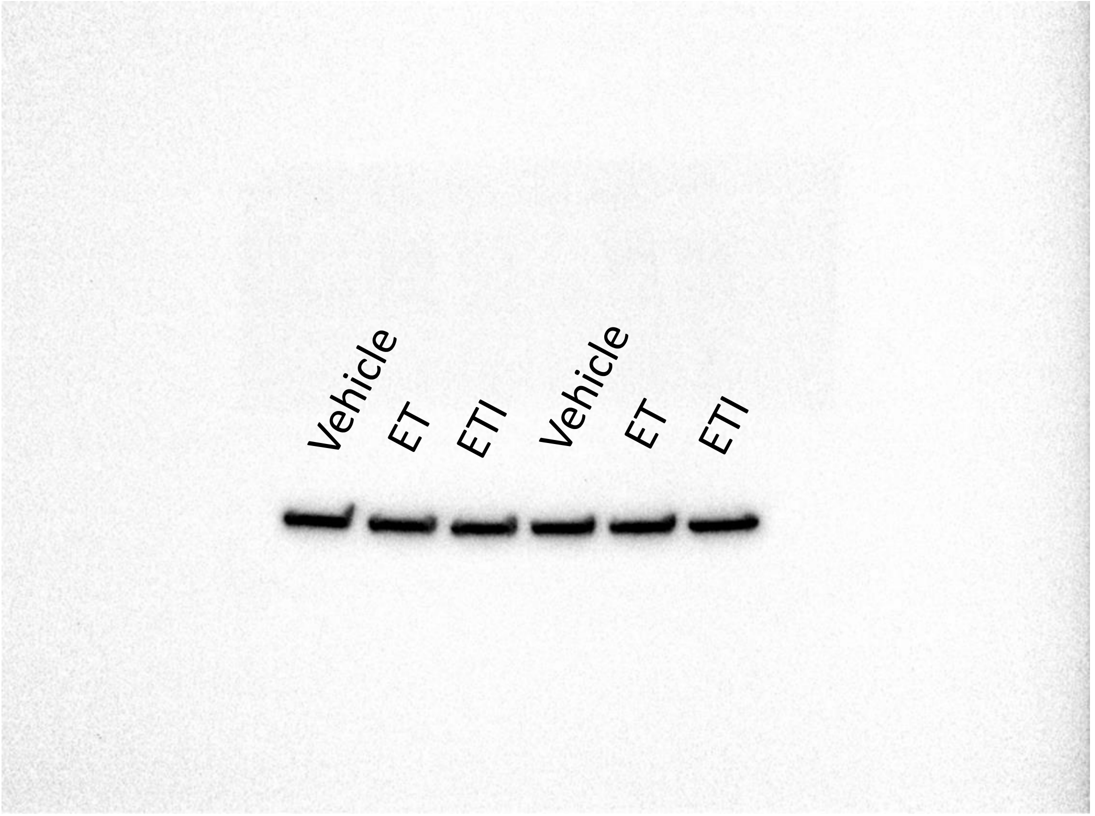

Blot III.

CFTR antibody 596

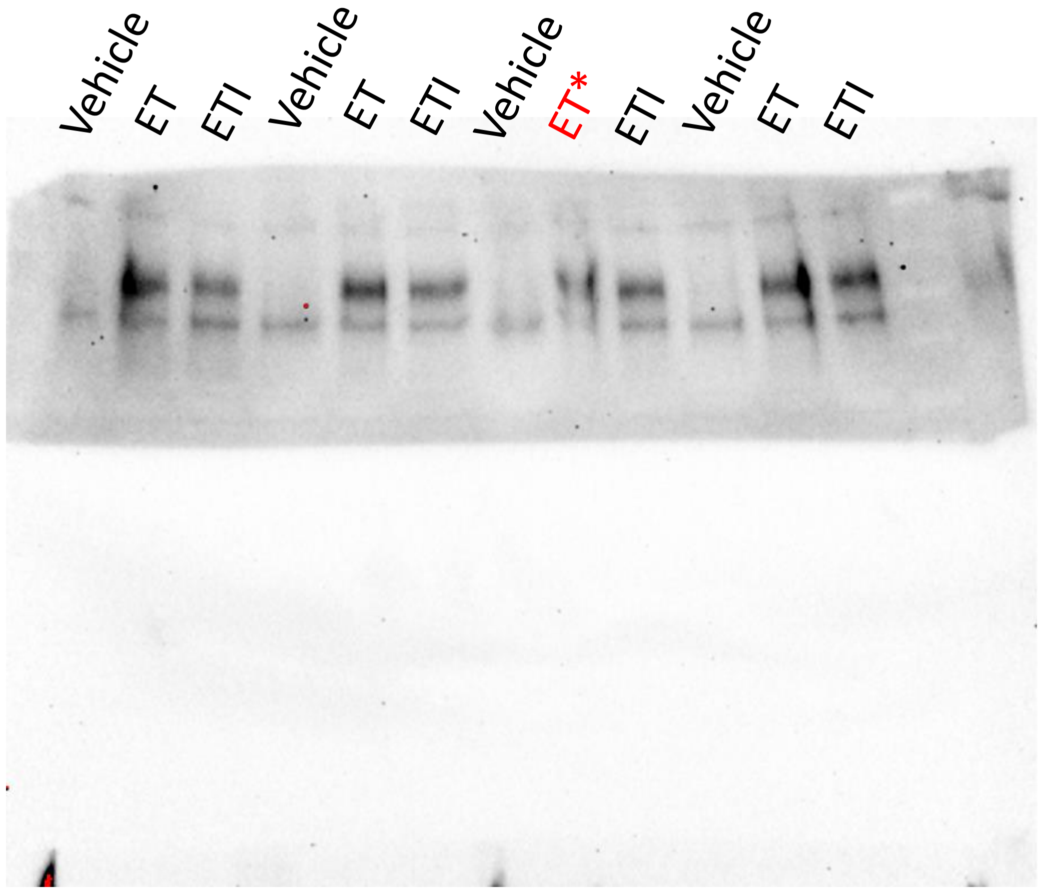

β-actin antibody

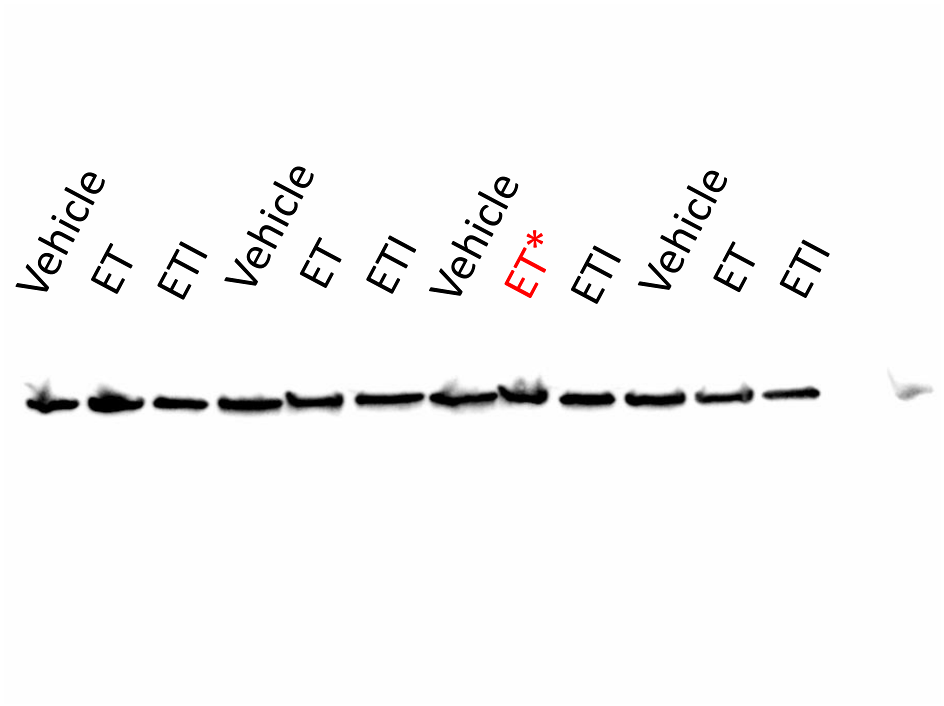

*excluded from analysis

Blot IV

CFTR antibody 596

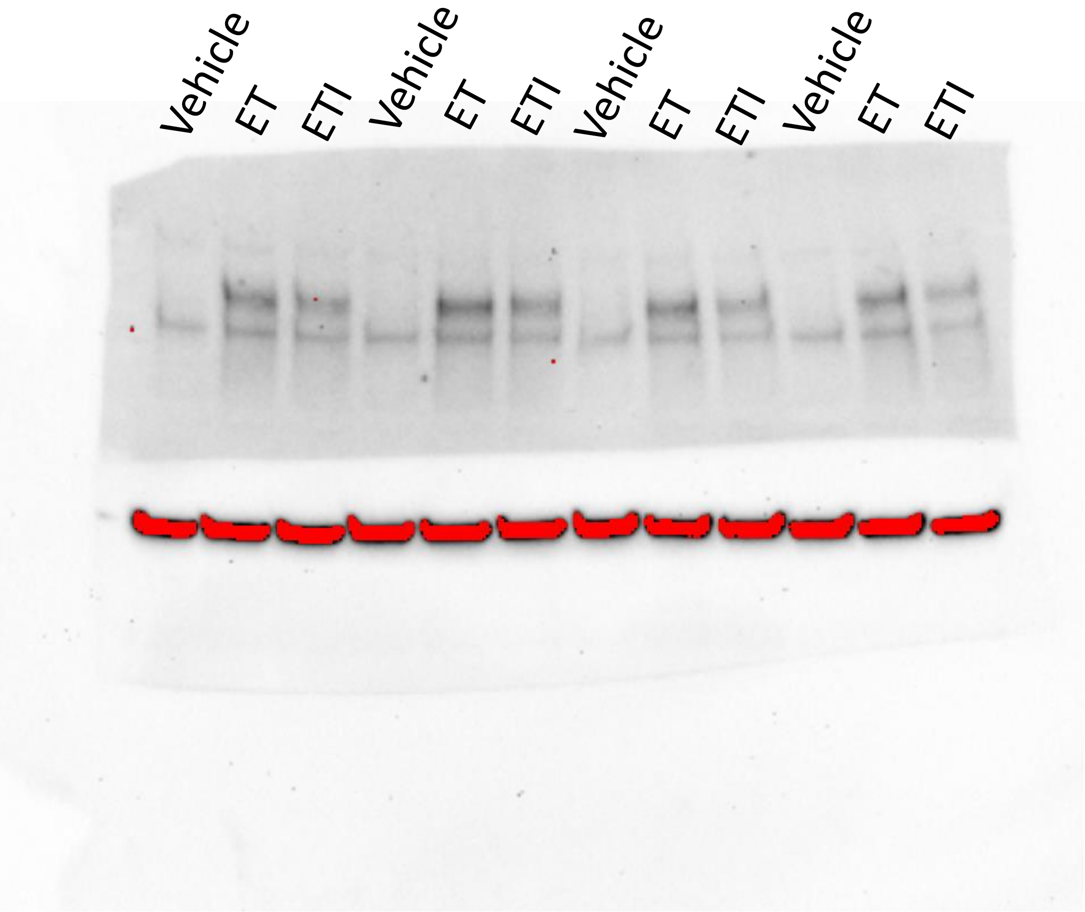

β-actin antibody

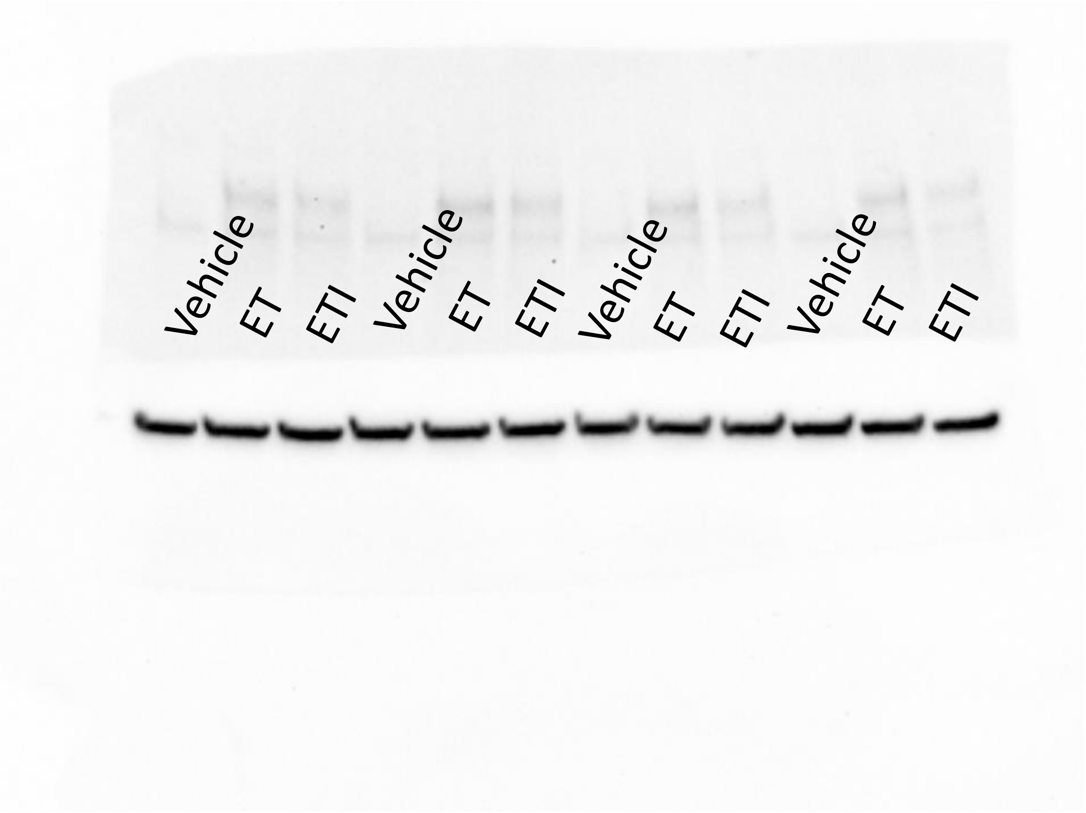

Supplement: Unedited blot and gel images [file jciinsight-10-187951-s052.pdf]
